# Tripartite motif 32 prevents pathological cardiac hypertrophy

**DOI:** 10.1042/CS20150619

**Published:** 2016-04-01

**Authors:** Lijuan Chen, Jia Huang, Yanxiao Ji, Xiaojing Zhang, Pixiao Wang, Keqiong Deng, Xi Jiang, Genshan Ma, Hongliang Li

**Affiliations:** * Department of Cardiology, Zhongda Hospital Affiliated to Southeast University, Jiangsu 210009, P.R. China; † Animal Experiment Center/Animal Biosafety Level-III Laboratory, Wuhan University, Wuhan, 430072, P.R. China

**Keywords:** Akt, cardiac dysfunction, cardiac hypertrophy, signalling pathway, TRIM32

## Abstract

TRIM32 (tripartite motif 32) is widely accepted to be an E3 ligase that interacts with and eventually ubiquitylates multiple substrates. TRIM32 mutants have been associated with LGMD-2H (limb girdle muscular dystrophy 2H). However, whether TRIM32 is involved in cardiac hypertrophy induced by biomechanical stresses and neurohumoral mediators remains unclear. We generated mice and isolated NRCMs (neonatal rat cardiomyocytes) that overexpressed or were deficient in TRIM32 to investigate the effect of TRIM32 on AB (aortic banding) or AngII (angiotensin II)-mediated cardiac hypertrophy. Echocardiography and both pathological and molecular analyses were used to determine the extent of cardiac hypertrophy and subsequent fibrosis. Our results showed that overexpression of TRIM32 in the heart significantly alleviated the hypertrophic response induced by pressure overload, whereas TRIM32 deficiency dramatically aggravated pathological cardiac remodelling. Similar results were also found in cultured NRCMs incubated with AngII. Mechanistically, the present study suggests that TRIM32 exerts cardioprotective action by interruption of Akt- but not MAPK (mitogen-dependent protein kinase)-dependent signalling pathways. Additionally, inactivation of Akt by LY294002 offset the exacerbated hypertrophic response induced by AB in TRIM32-deficient mice. In conclusion, the present study indicates that TRIM32 plays a protective role in AB-induced pathological cardiac remodelling by blocking Akt-dependent signalling. Therefore TRIM32 could be a novel therapeutic target for the prevention of cardiac hypertrophy and heart failure.

## Abstract (precis)

This study presents the first evidence that TRIM32 protects against pathological cardiac hypertrophy by suppressing Akt-dependent signalling pathways. Therefore TRIM32 might be a potential therapeutic strategy for the prevention and treatment of cardiac hypertrophy and heart failure.

## CLINICAL PERSPECTIVES

• The present study is the first to demonstrate that TRIM32 protects against pathological cardiac hypertrophy by repressing Akt-dependent signalling pathways.
• TRIM32 expression is decreased in the development of pathological cardiac hypertrophy and heart failure.
• These findings broaden our understanding of the molecular mechanisms of cardiac hypertrophy, and may provide novel strategies for the treatment of cardiac hypertrophy and heart failure.

## INTRODUCTION

Cardiac hypertrophy is the compensatory response of the heart to various stresses, including pressure or volume overload and ischaemia, and is characterized by increased weight and volume of cardiomyocytes with subsequent interstitial fibrosis [1,2]. However, the progression of cardiac hypertrophy leads to an elevated incidence of adverse cardiac events such as arrhythmias, sudden death and heart failure [3,4]. Extracellular stimuli, including biomechanical stress and neurohumoral mediators, are sensed by various receptors and converge on a limited number of intracellular signalling pathways [5]. Activation or inhibition of these signalling pathways eventually results in pathological cardiac hypertrophy though the regulation of multiple transcription factors that alter gene expression, increasing the rate of protein translation and decreasing the rate of protein degradation [5,6]. Therefore molecules that selectively modulate the activity of specific signalling pathways could potentially be therapeutic targets for the prevention of cardiac hypertrophy.

TRIM32 (tripartite motif 32) was first identified by Fridell et al. [7] as a protein that interacts with the activation domain of lentiviral Tat proteins. The *TRIM32* gene is located at chromosome 9q33.1 and encodes a 653-amino-acid protein that is ubiquitously expressed in adult tissues, but TRIM32 levels appear much higher in the brain and heart [8]. In addition to the common tripartite motif (RING, B-box, coiled coil) of the TRIM family, TRIM32 has six NHL repeats with putative protein-binding properties at the C-terminus. To date, seven mutations in the NHL repeats have been associated with LGMD-2H (limb girdle muscular dystrophy 2H) [9–13]. Moreover, Chiang et al. [14] identified a novel mutation in the B-box domain of TRIM32 resulting in BBS11 (Bardet–Biedl syndrome type 11), a pleiotropic disorder characterized by obesity, retinal dystrophy, polydactyly, and renal and cardiac abnormalities. Furthermore, owing to the presence of RING domains, TRIM32 has been reported to function as an E3 ligase that interacts with and eventually ubiquitylates multiple substrates, including c-Myc [15], PIASy [protein inhibitor of activated STAT (signal transducer and activator of transcription) y] [16], Abi-2 [17], XIAP (X-linked inhibitor of apoptosis) [18], dysbindin [19] and desmin [20], and participates in various pathological processes ranging from cancer to muscular dystrophy. Interestingly, upon fasting, TRIM32 interacts with myosin and desmin in skeletal muscle cells, leading to the ubiquitylation and degradation of desmin, actin, tropomyosin, troponins and actinin, which are components of the desmin filament, the thin filament or the Z-band [20]. Furthermore, Cohen et al. [21] reported that TRIM32 inhibits PI3K (phosphoinositide 3-kinase)/Akt signalling leading to muscle atrophy by promoting plakoglobin–PI3K dissociation. These results suggest that TRIM32 could confer protection against excessive tissue growth, especially in skeletal muscle. Given the similarity between skeletal muscle cells and cardiomyocytes, it is tempting to speculate that TRIM32 would be cardioprotective during cardiac hypertrophy.

In the present study, we provide the first evidence for an anti-hypertrophic role for TRIM32 in the heart. We show that the expression of TRIM32 decreases in human hearts with DCM (dilated cardiomyopathy). Moreover, TRIM32 was down-regulated in both mouse hearts and cardiomyocytes subjected to hypertrophic stresses. In addition, mice and isolated NRCMs (neonatal rat cardiomyocytes) with either overexpression or deletion of TRIM32 were generated to investigate the role and underlying mechanisms of TRIM32 in cardiac hypertrophy. We provide evidence that TRIM32 overexpression attenuates cardiac hypertrophy and subsequent fibrosis by repressing PI3K/Akt signalling, whereas TRIM32 deficiency conferred the opposite phenotype in response to myocardial pressure overload. Taken together, our results identify TRIM32 as a novel negative regulator of pathological cardiac hypertrophy, mainly through its suppressive action on PI3K/Akt signalling.

## MATERIALS AND METHODS

### Reagents

The antibodies against ANP (atrial natriuretic peptide) (sc20158) and β-MHC (β-myosin heavy chain) (sc53090) were from Santa Cruz Biotechnology. The antibody against TRIM32-(28–984) was from ProSci. The antibody against GAPDH (glyceraldehyde-3-phosphate dehydrogenase) (MB001) was from Bioworld Technology. The antibodies against p-MEK1/2 [MAPK (mitogen-activated protein kinase)/ERK (extracellular-signal-regulated kinase) kinase 1/2] (9154), total MEK1/2 (9122), p-ERK1/2 (4370), total ERK1/2 (4695), p-JNK1/2 (c-Jun N-terminal kinase 1/2) (4668), total JNK1/2 (9258), p-p38 (4511), total p38 (9212), p-Akt (4060), total Akt (4691), p-GSK3β (glycogen synthase kinase 3β) (9322), total GSK3β (9315), p-mTOR (mammalian target of rapamycin) (2971), total mTOR (2983) and total p70 (2708) were from Cell Signaling Technology. The antibody against p-p70 was from GeneTex (GTX50304). The BCA protein assay kit was from Pierce. FBS was from Hyclone. Cell culture reagents and all other reagents were from Sigma.

### Human ventricular samples

Samples of failing human hearts were obtained from the LVs (left ventricles) of DCM patients undergoing heart transplantation for end-stage heart failure. The control samples were obtained from normal heart donors who had died from accidents unrelated to cardiac reasons, but whose hearts were unsuitable for transplantation for technical reasons. Written informed consent was obtained from DCM patients undergoing heart transplantation and the families of prospective heart donors. All procedures involving human samples conformed to the principles outlined in the Declaration of Helsinki and were approved by the Ethics Committee at Zhongda Hospital affiliated to Southeast University in Nanjing, China.

### Study animals

All experiments involving animals were approved by the Animal Care and Use Committee of Zhongda Hospital of Southeast University. The following animals were used.

#### Production of TRIM32-global-KO (knockout) mice

The online CRISPR (clustered regularly interspaced short palindromic repeats) design tool was used to predict the guiding sequences aiming at the target site of the *Trim32* gene in the mouse genome (http://crispr.mit.edu). A pair of oligomers (oligo1, 5′-TAGGCTCCAGACACTGGCGGCAGA-3′, and oligo2, 5′-AAACTCTGCCGCCAGTGTCTGGAG-3′) were constructed in the BsaI restriction site of the pUC57-sgRNA expression vector (Addgene). DNA was amplified by PCR using primers (forward primer, 5′-GATCCCTAATACGACTCACTATAG-3′, and reverse primer, 5′-AAAAAAAGCACCGACTCGGT-3′) spanning the sgRNA (single guide RNA) regions and the T7 promoter. After purification, sgRNA was transcribed with the MEGA shortscript Kit (Ambion) and purified using an miRNeasy Micro Kit (Qiagen). The Cas9 (CRISPR-associated 9) expression plasmid (Addgene) was linearized by PmeI and subsequently used as the template for *in vitro* transcription with the T7 Ultra Kit (Ambion). The RNeasy Mini Kit (Qiagen) was used to purify mRNA according to the manufacturer's instructions. Then, Cas9 and sgRNA mRNA injections for one-cell embryos were performed with the FemtoJet 5247 microinjection system under standard conditions. Genomic DNA was extracted from mouse tails using phenol/chloroform and alcohol precipitation. A 336-bp DNA fragment including the sgRNA target site was amplified by PCR using the following primers: TRIM32-forward (5′-GGAATCTGACACTGGGGCAT-3′) and TRIM32-reverse (5′-TGATCTTCAGCACCGTCAGG-3′). The purified PCR product was denatured and reannealed in NEB Buffer 2 to create heteroduplex DNA which was then digested by T7EN (NEB) for 45 min and subsequently determined on a 2.0% agarose gel. Each mouse was sequenced to identify frameshift mutations. F1 and F2 offspring were screened with the primers TRIM32-F (5′-CCATCTGCATGGAGTCCTTC-3′) and TRIM32-R (5′-AGGCTGGTGATGCGAGTAAT-3′). Subsequently, PCR products were determined using 2.0% agarose gel electrophoresis.

#### Production of TG (transgenic) mice

The transgene vector pCAG-CAT-TRIM32, which contains a CAG gene promoter–loxP–CAT gene–loxP–TRIM32 region, was constructed from pCAG-loxP-CAT-loxP-lacZ by replacing the *lacZ* gene with mouse *Trim32* cDNA. The construct was linearized and purified using the QIAquick Gel Extraction Kit (Qiagen), following the manufacturer's instructions, and was subsequently used for pronuclear microinjection. Founder TG mice were identified by tail DNA amplification and were then bred with C57BL/6J mice. The PCR primers for transgene detection were CAG gene promoter-forward (5′-CCCCCTGAACCTGAAACATA-3′) and TRIM32-reverse (5′-AATGATCTTCAGCACCGTCA-3′) and yielded a 566-bp product. CAG-CAT-TRIM32 mice (strain C57BL/6J) were mated with α-MHC-MerCreMer (Jackson Laboratory) mice to generate CAG-CAT-TRIM32/α-MHC-MerCreMer double-TG mice. Then, double-TG mice were injected with tamoxifen (80 mg/kg per day) for 5 days at 6 weeks of age to generate cardiac-specific conditional TRIM32-TG mice. The α-MHC-MerCreMer mice with tamoxifen administration (MCT) were used as the control group.

### AB (aortic banding)

AB [22,23] was applied to establish a pressure overload-induced cardiac hypertrophy model. Male mice aged 8–10 weeks at 24–27 g of body weights were subjected to AB surgery, and all experiments and subsequent analyses were conducted in a blinded fashion. Before surgery, the mice were anaesthetized with pentobarbital sodium (50 mg/kg, intraperitoneal injection; Sigma). When the toe-pinch reflex was absent, the left chest of each mouse was opened to identify the thoracic aorta by blunt dissection at the second intercostal space. Then, we performed AB with 7-0 silk sutures by banding the descending aorta (thoracic aorta) with a 26/27-gauge needle. The needle was removed before closing the thoracic cavity. We also utilized Doppler echocardiography to confirm that the constriction of the aorta was adequate. In contrast, sham-operated mice underwent the same surgical procedure without constricting the aorta. In parallel with AB surgeries, the specific Akt inhibitor LY294002 (Sigma) dissolved in DMSO was administered intraperitoneally to WT (wild-type) and TRIM32-KO mice (50 mg/kg) daily for 4 weeks. This dose was determined by a preliminary dose-ranging study from 0 to 100 mg of LY294002/kg of body weight (intraperitoneal injection) in which 50 mg/kg was found to yield significant inhibition of Akt activation *in vivo*. The control group in these experiments was administered with the same volume of DMSO.

### Echocardiography measurements

Echocardiography measurements were performed at the indicated times after each mouse was anaesthetized with 1.5–2% isoflurane. Echocardiography was performed by SONOS 5500 ultrasound (Philips Electronics) with a 15-MHz linear array ultrasound transducer. The LV was measured in both the parasternal long-axis and short-axis views at a frame rate of 120 Hz. End-systole or end-diastole were considered as the phase when the areas of the LV were the smallest or largest respectively. The LVESD (LV end-systolic diameter) and LVEDD (LV end-diastolic diameter) at the mid-papillary muscle level were obtained by M-mode tracing with a sweep speed of 50 mm/s. The percentage of left ventricular FS (fractional shortening) was defined as (LVEDD−LVESD)/LVEDD×100%. More than five individual mice per group were measured by the M-mode tracing in triplicate.

### Histological analysis

Hearts were excised and washed with saline solution. After being fixed in 10% formalin, they were embedded in paraffin using standard histological procedures. Subsequently, hearts were cut transversely close to the apex to visualize the left and right ventricles. Several sections (5-μm-thick) obtained from the mid-papillary muscle level of each heart were stained with H&E (haematoxylin and eosin) to assess histopathology or with PSR (Picrosirius Red) to evaluate collagen deposition. In addition, sections were stained with FITC-conjugated WGA (wheatgerm agglutinin) (Invitrogen) to determine the cross-sectional area of the myocytes. Images of single myocytes and collagen deposition were captured by microscopy and measured with a quantitative digital analysis imaging system (Image-Pro Plus 6.0). More than 100 oval-shaped myocytes in the sections from at least five different mouse samples were calculated in each group.

### Cultured NRCMs and recombinant adenoviral vectors

Primary cultures of NRCMs were performed as described previously [24,25]. Neonatal Sprague–Dawley rats were killed by swift decapitation in accordance with the Guide for the Care and Use of Laboratory Animals published by the U.S. National Institutes of Health. Then, ventricular myocytes were isolated and seeded in six-well culture plates coated with gelatin at a density of 3×10^5^ cells/well in DMEM (Dulbecco's modified Eagle's medium)/Ham's F12 medium including 10% (v/v) FBS, BrdU (bromodeoxyuridine) and penicillin/streptomycin. After 48 h, the culture medium was replaced by serum-free DMEM/Ham's F12 for 12 h and then stimulated with AngII (angiotensin II) (1 μmol/l) or PBS. To overexpress TRIM32, the entire coding region of the rat *Trim32* gene, under control of the cytomegalovirus promoter, was inserted in replication-defective adenoviral vectors. A similar adenoviral vector encoding the *GFP* gene (AdGFP) was used as a control. To knockdown TRIM32 expression, three rat shTRIM32 constructs were purchased from SABiosciences (KR43343G). Subsequently, three AdshTRIM32 (adenoviral vector encoding the TRIM32 shRNA sequence) adenoviruses were generated. Among them, the construct that repressed the expression of TRIM32 to the greatest extent was selected for all further experiments. AdshRNA (adenoviral vector encoding scrambled shRNA sequence) was applied as a control. NCRMs were infected with different adenovirus in diluted medium at a multiplicity of infection of 100 for 24 h.

### Total RNA isolation and quantitative real-time PCR

TRIzol® reagent (Invitrogen) was used to extract total RNA from LV tissues and cultured cardiomyocytes as described by the manufacturer. Then, RNA was reverse-transcribed into cDNA using the Transcriptor First Strand cDNA Synthesis Kit (Roche). Quantitative real-time PCR amplification was performed in the SYBR Green PCR Master Mix (Applied Biosystems). Each PCR was performed in triplicate and the results are expressed as the average of relative gene expression normalized to *GAPDH* gene expression.

### Western blotting

LV tissues and cultured cardiomyocytes were first lysed in lysis buffer (720 μl of RIPA, 20 μl of PMSF, 100 μl of Complete protease inhibitor, 100 μl of Phos-stop, 50 μl of NaF and 10 μl of Na_3_VO_4_). Soluble extracts were incubated on ice for 15 min, followed by centrifugation at 14000 ***g*** for 30 min at 4°C. After centrifugation of the samples, the protein concentrations were determined using the BCA Protein Assay Kit (Pierce). The lysates (50 μg) were separated by SDS/PAGE (Invitrogen) and transferred on to a PVDF membrane (Millipore). After blocking with 5% (w/v) non-fat dried skimmed milk powder for 1 h at room temperature, membranes were incubated with the specific primary antibodies overnight at 4°C. After incubation with the horseradish peroxidase-conjugated secondary antibodies for 1 h at room temperature, immunoblots were revealed by the ChemiDoc™ XRS^+^ (Bio-Rad Laboratories). The expression levels of specific proteins were normalized to GAPDH on the same membrane.

### Statistical analysis

The data are presented as means±S.D. Comparisons between two groups were determined using an unpaired Student's *t* test, whereas one-way ANOVA followed by Bonferroni's post-hoc test (assuming equal variances) or Tamhane's T2 post-hoc test (without the assumption of equal variances) was applied for comparisons among more than two groups. All statistical analyses were performed with SPSS software, version 19.0, and *P*<0.05 was considered statistically significant.

## RESULTS

### TRIM32 expression is decreased in failing human hearts and hypertrophic mouse hearts

**Figure 1:**
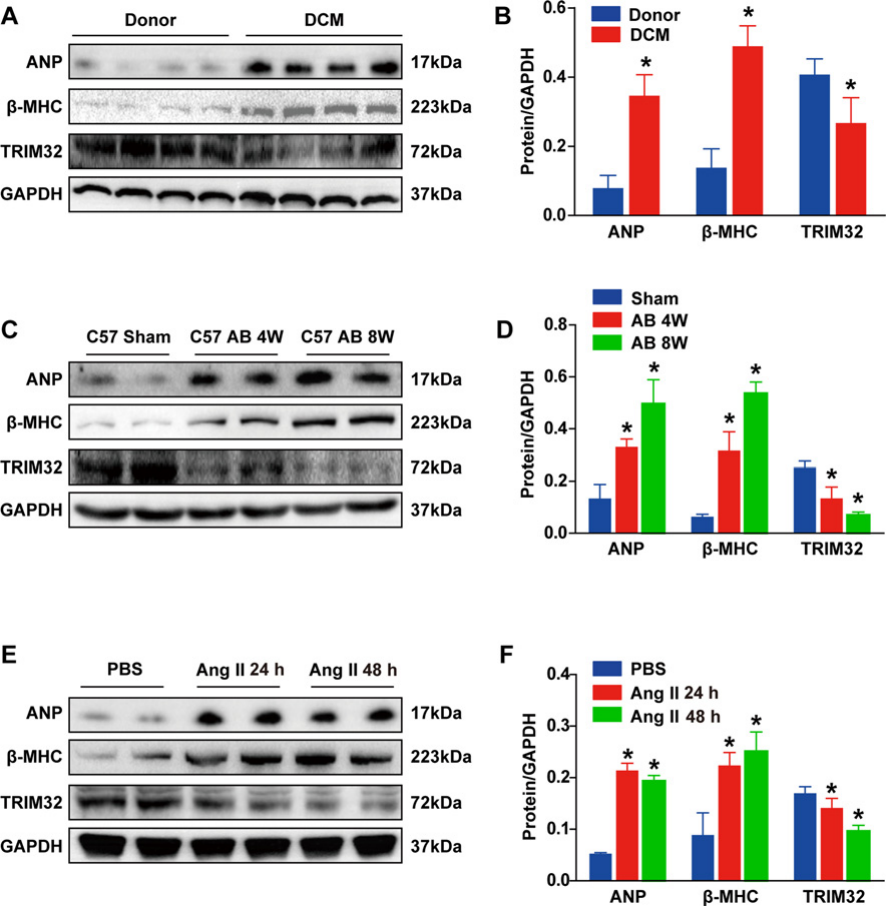
TRIM32 expression is decreased in failing human hearts and hypertrophic mouse hearts (**A** and **B**) Representative Western blots (**A**) and quantitative results (**B**) of ANP, β-MHC and TRIM32 protein expression in normal donor hearts and in human hearts with DCM (*n*=8 hearts per experimental group; *P<0.05 compared with donor hearts). (**C** and **D**) Representative Western blots (**C**) and quantitative results (**D**) of ANP, β-MHC and TRIM32 protein expression in a hypertrophic mouse heart induced by AB for the times indicated (W, weeks) (*n*=6 mice per experimental group; *P<0.05 compared with sham). (**E** and **F**) Representative Western blots (**E**) and quantitative results (**F**) of ANP, β-MHC and TRIM32 protein expression in cultured NRCMs treated with AngII for 24 or 48 h (*n*=4 samples per experimental group; four independent experiments; *P<0.05 compared with PBS). Results are means±S.D., and statistical analyses were determined using an unpaired Student's *t* test. Molecular masses are indicated in kDa in the Western blot panels.

To study the potential role of TRIM32 in the development of cardiac hypertrophy and heart failure, we first assessed whether cardiac TRIM32 expression levels were altered in failing human heart samples. In comparison with normal donor hearts, the amount of TRIM32 protein, as determined by Western blot assays, was dramatically decreased in failing human hearts. Meanwhile, the reduction in TRIM32 expression was paralleled by an increased expression of cardiac hypertrophic markers, including ANP and β-MHC (Figures 1A and 1B). Furthermore, in AB-induced hypertrophic mouse hearts, as was evident by progressive up-regulation of ANP and β-MHC expression, TRIM32 levels were significantly down-regulated by approximately 47% at 4 weeks after AB and by approximately 72% at 8 weeks after AB, compared with sham-treated hearts (Figures 1C and 1D). In addition, after treating cultured NRCMs with AngII (1 μmol/l) to induce cardiomyocyte hypertrophy *in vitro*, Western blot assays revealed that TRIM32 levels were progressively decreased in AngII-treated samples, compared with PBS treatment (Figures 1E and 1F). Altogether, these data suggest that TRIM32 deficiency correlates with the development of cardiac hypertrophy and heart failure.

### TRIM32 attenuates AngII-induced cardiomyocytes hypertrophy *in vitro*

**Figure 2:**
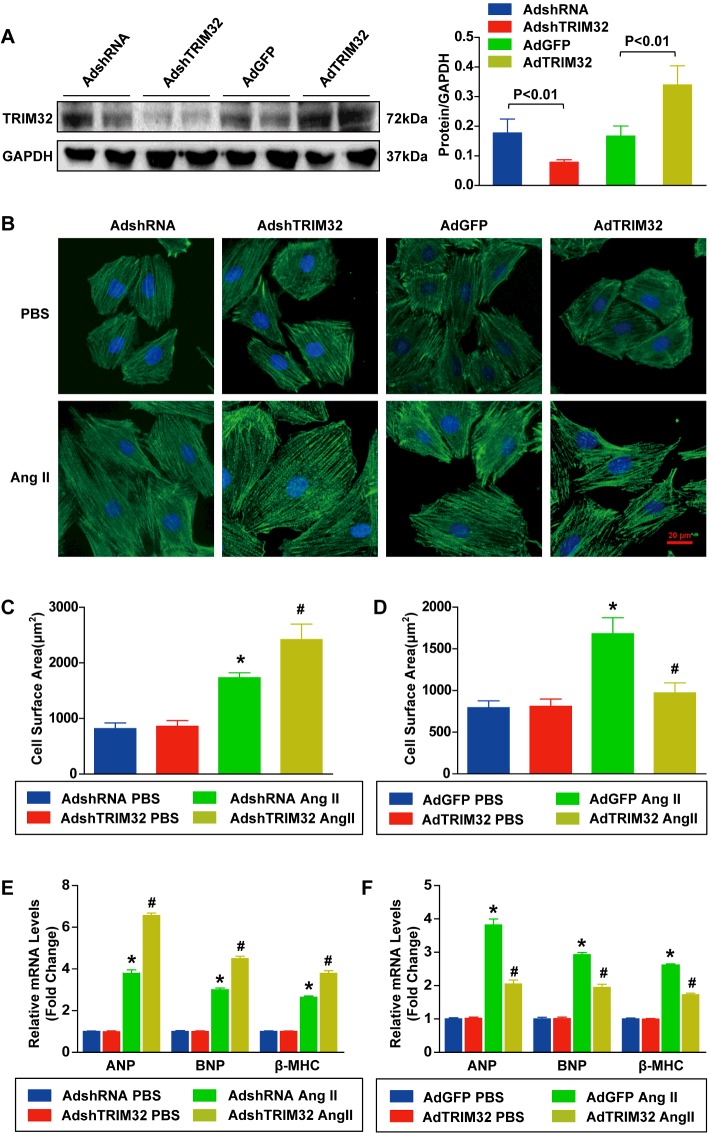
TRIM32 attenuates AngII-induced cardiomyocyte hypertrophy *in vitro* (**A**) The protein expression level of TRIM32 in NRCMs after infection with AdshRNA, AdshTRIM32, AdGFP or AdTRIM32 was determined by Western blotting (*n*=4 samples per experimental group). Left: representative blots. Right: quantitative results. (**B**) Representative images of NRCMs infected with AdshRNA, AdshTRIM32, AdGFP or AdTRIM32 and treated with AngII (1 μmol/l) or PBS for 48 h (*n*=4 samples per experimental group; blue, nucleus; green, α-actinin; scale bar, 20 μm). (**C**) Quantitative results of the cell surface area of NRCMs infected with AdshTRIM32 or AdshRNA subjected to AngII (1 μmol/l) or PBS for 48 h (*n*≥50 cells per experimental group; *P<0.05 compared with AdshRNA/PBS; #*P*<0.05 compared with AdshRNA/AngII). (**D**) Quantitative results of the cell surface area of NRCMs infected with AdTRIM32 or AdGFP subjected to AngII (1 μmol/l) or PBS for 48 h (*n*≥50 cells per experimental group; *P<0.05 compared with AdGFP/PBS; #*P*<0.05 compared with AdGFP/AngII). (**E**) Relative mRNA levels of ANP, BNP and β-MHC in AdshTRIM32-infected NRCMs and AdshRNA-infected NRCMs after treatment with PBS or AngII (1 μmol/l) for 48 h were determined by real-time PCR assays (*n*=4 samples per experimental group; *P<0.05 compared with AdshRNA/PBS; #*P*<0.05 compared with AdshRNA/AngII). (**F**) Relative mRNA levels of ANP, BNP and β-MHC in AdTRIM32-infected NRCMs and AdGFP-infected NRCMs after treatment with PBS or AngII (1 μmol/l) for 48 h were determined by real-time PCR assays (*n*=4 samples per experimental group; *P<0.05 compared with AdGFP/PBS; #*P*<0.05 compared with AdGFP/AngII). Results are means±S.D. from four independent experiments, and statistical analyses were performed using one-way ANOVA.

As TRIM32 expression was decreased during the development of cardiac hypertrophy and heart failure, we investigated whether TRIM32 could modulate the progression of cardiac hypertrophy. We generated NRCMs with either reduced or elevated levels of TRIM32 by infection with AdshTRIM32 or AdTRIM32 (adenoviral vector encoding *TRIM32* cDNA) respectively (Figure 2A). Then, the cardiomyocytes were administered with either AngII (1 μmol/l) or PBS as a control for 48 h before immunostaining for α-actinin to determine cardiomyocyte surface area. With the administration of PBS, there were no significant differences in cardiomyocyte morphology or cardiomyocyte surface area between each group. However, compared with AdshRNA-infected controls, AdshTRIM32 infection-mediated TRIM32 deficiency dramatically exaggerated AngII-induced cardiomyocyte hypertrophy, as was evident by up-regulation of cardiomyocyte surface area (2419±280 compared with 1734±88 μm^2^; *P*<0.05; Figures 2B and 2C). Conversely, AdTRIM32 infection-mediated TRIM32 overexpression markedly attenuated AngII-induced cardiomyocyte hypertrophy compared with AdGFP-infected controls (972±120 compared with 1681±191 μm^2^; *P*<0.05; Figures 2B and 2D). Similarly, in response to AngII, TRIM32-deficient cardiomyocytes had greater mRNA expression of cardiac hypertrophic markers including ANP, BNP (brain natriuretic peptide) and β-MHC, whereas TRIM32-overexpressing cardiomyocytes had lower expression compared with controls (Figures 2E and 2F). These *in vitro* data indicate that TRIM32 may act as a negative regulator of pathological cardiac hypertrophy.

### TRIM32 overexpression attenuates pressure overload-induced cardiac hypertrophy and heart failure

**Figure 3:**
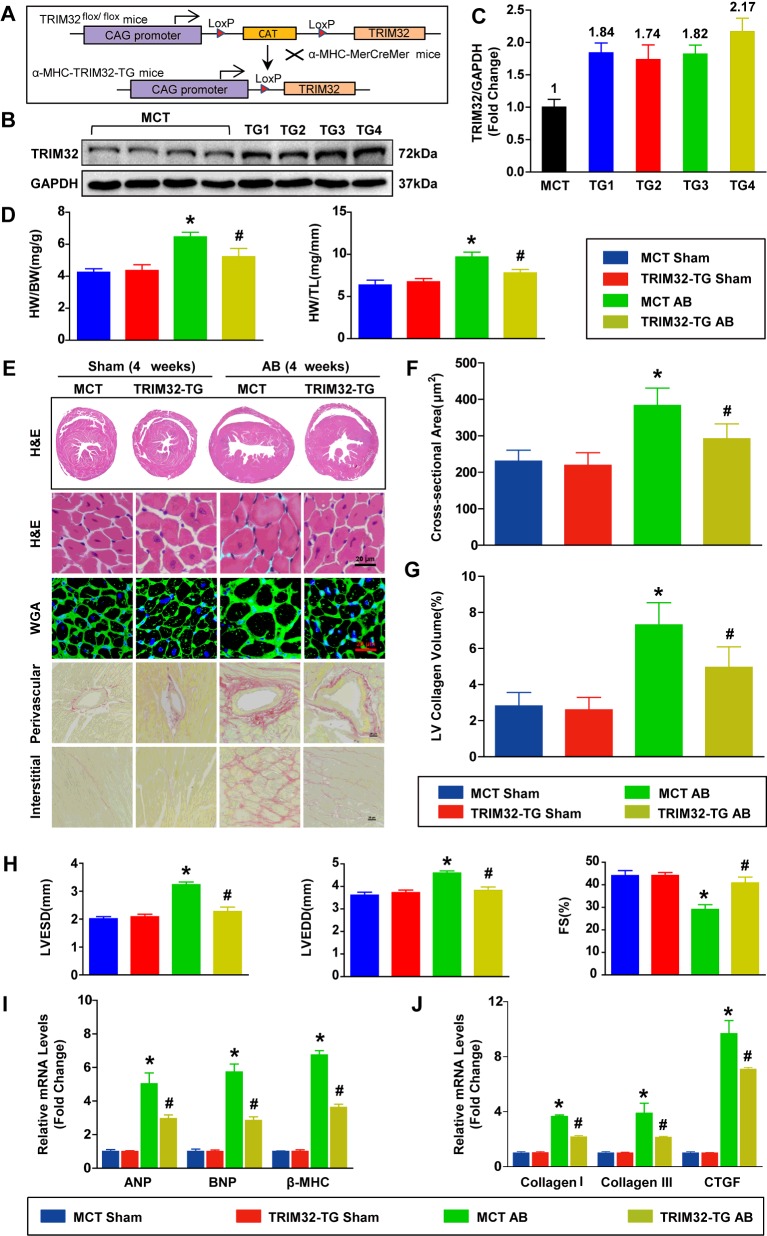
TRIM32 overexpression attenuates pressure overload-induced cardiac hypertrophy and heart failure (**A**) Schematic diagram of the construction of TG mice with a full-length murine *Trim32* cDNA under the control of the α-MHC promoter. (**B** and **C**) Representative Western blots (**B**) and quantitative results (**C**) of TRIM32 protein expression in heart tissue from four TG lines and MCT mice (*n*=3 mice per experimental group; three independent experiments). Molecular masses are indicated in kDa in (**B**). (**D**) Statistical results for the ratios of HW/BW and HW/TL in MCT mice and TRIM32-TG mice at 4 weeks after sham or AB (*n*=8–10 mice per experimental group). (**E**) Representative images of gross morphology, H&E staining, WGA staining and PSR staining of hearts from the indicated groups (*n*=5 mice per experimental group; scale bar, 20 μm for lower H&E staining, WGA staining and PSR staining). (**F**) Statistical results for the cardiomyocyte cross-sectional area in the indicated groups (*n*≥100 cells per experimental group). (**G**) Quantification of left ventricular collagen volume in the indicated groups (*n*≥25 fields per experimental group). (**H**) Measurements of LVESD, LVEDD and FS in the indicated groups (*n*=8–10 mice per experimental group). (**I**) Relative mRNA levels of ANP, BNP and β-MHC in the indicated groups were determined by real-time PCR assays (*n*=4 mice per experimental group). (**J**) Relative mRNA levels of collagen I, collagen III and CTGF in the indicated groups were determined by real-time PCR assays (*n*=4 mice per experimental group). *P<0.05 compared with MCT/sham; #*P*<0.05 compared with MCT/AB. Results are means±S.D., and statistical analyses were performed using one-way ANOVA.

To investigate whether compensatory overexpression of TRIM32 in hearts would alleviate pressure overload-induced cardiac hypertrophy and heart failure *in vivo*, we generated TG mice with cardiac-specific TRIM32 overexpression. A schematic diagram is shown in Figure 3(A), and detailed information is listed in the Materials and methods section. Four independent lines of TRIM32-TG mice (TG1, TG2, TG3 and TG4) were established and certified by Western blot analysis (Figures 3B and 3C). At baseline, all lines of TRIM32-TG mice were healthy and fertile, and there were no apparent abnormalities in their cardiac morphology or function compared with MCT mice (results not shown). Then we chose the TG4 line with the highest levels of TRIM32 expression in the heart for the following experiments. Subsequently, TRIM32-TG mice and MCT mice underwent AB surgery for 4 weeks. TRIM32 overexpression significantly repressed AB-mediated cardiac hypertrophy, as indicated by reduction in the ratios of heart weight to body weight (HW/BW), the ratios of HW to tibia length (HW/TL) (Figure 3D), the gross size of the heart (Figure 3E) and the cardiomyocyte cross-sectional area (Figure 3F) in TRIM32-TG mice compared with MCT mice. In addition, PSR staining was utilized to evaluate the extent of fibrosis. Both interstitial and perivascular fibrosis were markedly alleviated in TRIM32-TG hearts, but this was more pronounced in MCT hearts 4 weeks after AB (Figures 3E and 3G). TRIM32-TG mice consistently exhibited less ventricular dilation than MCT mice, as was evident by down-regulation of LVEDD and LVESD (Figure 3H). Similarly, echocardiography measurements showed significantly improvement in FS from TRIM32-TG mice compared with MCT mice, implying better cardiac contractility (Figure 3H). Accordingly, the mRNA expression levels of hypertrophic markers including ANP, BNP and β-MHC, as well as indices of collagen synthesis, including collagen I, collagen III and CTGF (connective tissue growth factor), were markedly increased in MCT hearts, but were attenuated in TRIM32-TG hearts after AB (Figure 3I and 3J). Altogether, these data support the concept that TRIM32 may protect the heart against hypertrophic stimuli.

### Generation of global TRIM32-deficient mice

**Figure 4:**
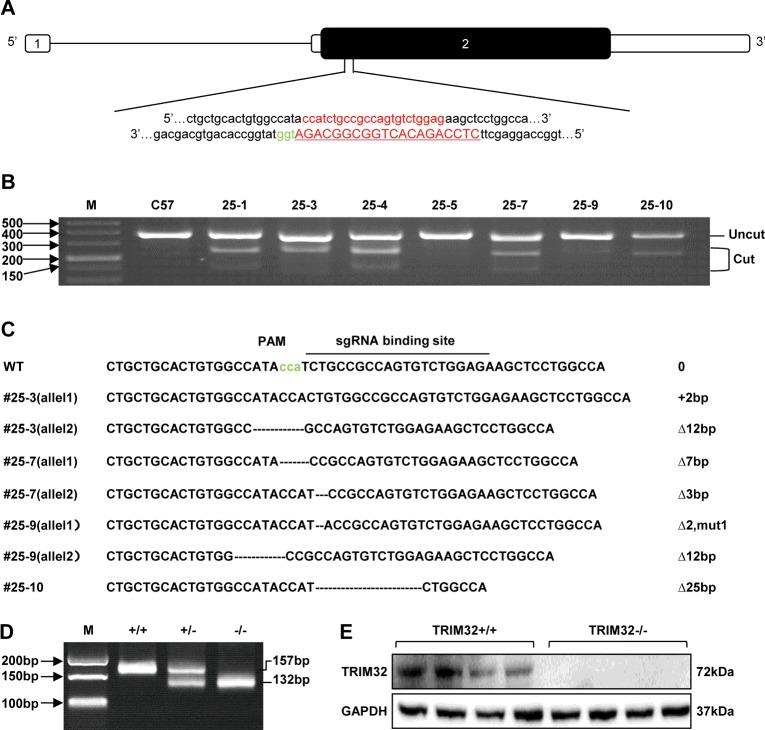
Generation of global TRIM32-deficient mice (**A**) One sgRNA targeted a region downstream of the 5′ end of exon 2 in the *Trim32* mouse gene. (**B**) Representative results of the T7E1 assay from pups subjected to microinjection. Sizes are indicated in bp. (**C**) Representative results of the DNA sequencing from mice with frameshift mutations. (**D**) Agarose gel photograph illustrating genotyping results of PCR products from WT (+/+), heterozygous (+/−) and TRIM32-KO (−/−) mice. Sizes are indicated in bp. (**E**) Representative Western blots of TRIM32 expression in heart tissues from global TRIM32-KO mice and their littermate controls (*n*=4 mice per experimental group). Molecular masses are indicated in kDa.

TRIM32-global-KO mice were generated to explore further the effect of TRIM32 deficiency on cardiac hypertrophy and heart failure *in vivo*. One sgRNA targeting downstream of the 5′-end of exon 2 of the mouse *Trim32* gene was designed and constructed (Figure 4A). After microinjection, the T7E1 assay found six of the total ten pups with cleavage products (Figure 4B), implying a mixture of WT and mutant DNA templates in these mice. Following subcloning of the PCR products from this animal, six subclones from each mouse were sequenced. Among them, only four (#25-3, #25-7, #25-9 and #25-10) indels produced frameshift mutations, but three (#25-3, #25-7 and #25-9) of them carried a double-mutant allele (Figure 4C). Then, founder #25-10 was mated with C57B6/J mice to generate heterozygous F1 offspring, which were interbred to establish the TRIM32-global-KO mouse strain (Figures 4D and 4E).

### TRIM32 deficiency aggravates pressure overload-induced cardiac hypertrophy and heart failure

**Figure 5:**
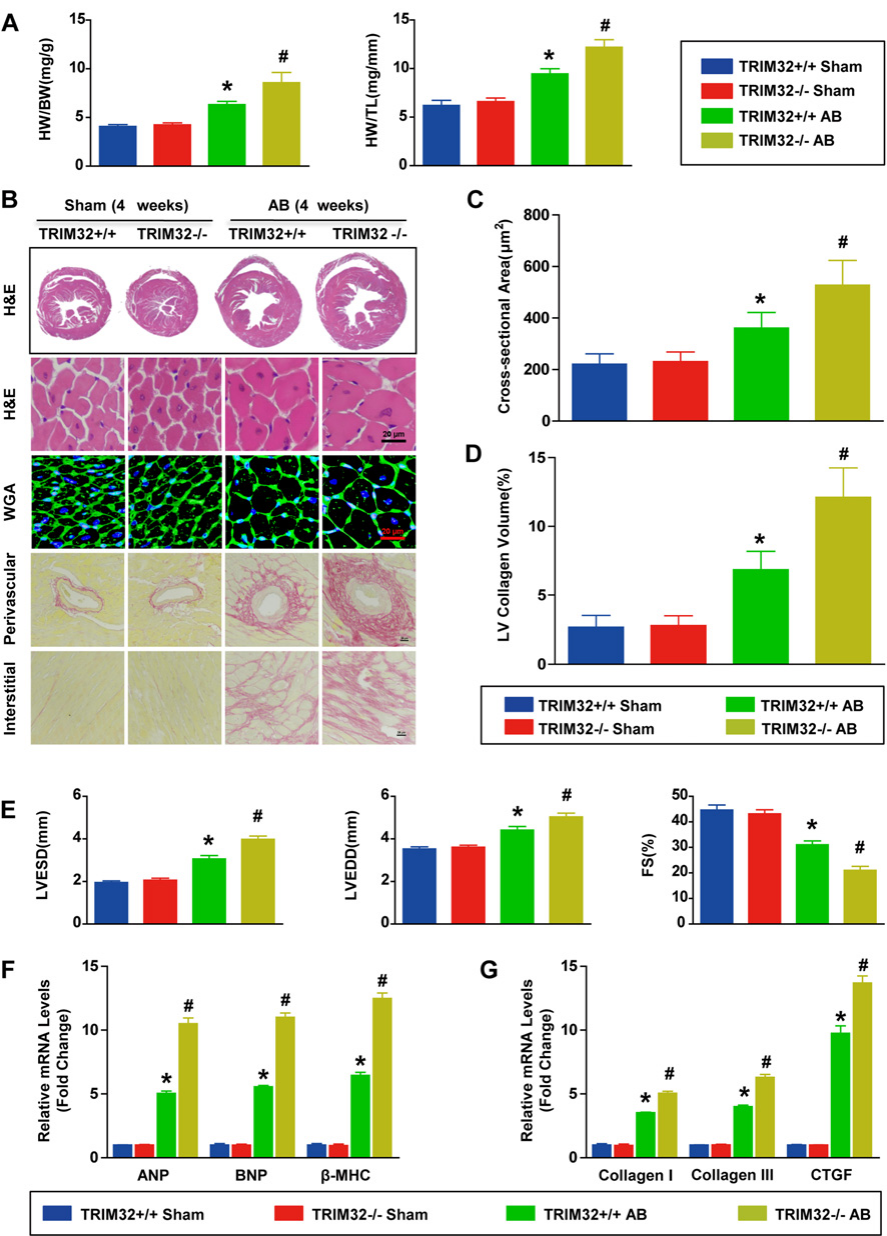
TRIM32 deficiency aggravates pressure overload-induced cardiac hypertrophy and heart failure (**A**) Statistical results for HW/BW and HW/TL in WT mice and TRIM32-KO mice at 4 weeks after sham or AB (*n*=8–10 mice per experimental group). (**B**) Representative images of gross morphology, H&E staining, WGA staining and PSR staining of hearts from the indicated groups (*n*=5 mice per experimental group; scale bar, 20 μm for lower H&E staining, WGA staining and PSR staining). (**C**) Statistical results for the cardiomyocyte cross-sectional area in the indicated groups (*n*≥100 cells per experimental group). (**D**) Quantification of left ventricular collagen volume in the indicated groups (*n*≥25 fields per experimental group). (**E**) Measurements of LVESD and LVEDD and FS in the indicated groups (*n*=8–10 mice per experimental group). (**F**) Relative mRNA levels of ANP, BNP and β-MHC in the indicated groups were determined by real-time PCR assays (*n*=4 mice per experimental group). (**G**) Relative mRNA levels of collagen I, collagen III, and CTGF in the indicated groups were determined by real-time PCR assays (*n*=4 mice per experimental group). *P<0.05 compared with WT/sham; #*P*<0.05 compared with WT/AB. Results are means±S.D., and statistical analyses were performed using one-way ANOVA.

Under basal conditions, the TRIM32-KO mice were healthy and fertile, and there were no apparent abnormalities in their cardiac morphology or function compared with WT mice (results not shown). In the present study, 4 weeks of AB led to an increase in HW/BW, HW/TL, the gross size of the heart and the cardiomyocyte cross-sectional area in WT mice; this induction was more significant in hearts from TRIM32-KO mice (Figures 5A–5C). Then we investigated the cardiac fibrosis secondary to cardiac hypertrophy. Sections of hearts were stained with PSR to evaluate the extent of fibrosis, which was quantified as the left ventricular collagen volume. At 4 weeks after AB, TRIM32-KO mice exhibited a more prominent cardiac interstitial and perivascular fibrosis compared with WT mice (Figures 5B and 5D). Consistent with deterioration of pathological cardiac hypertrophy, TRIM32-KO mice exhibited more significant ventricular dilation compared with WT mice, as indicated by further increase in LVEDD and LVESD (Figure 5E). In addition, largely reduced FS implying a decline in cardiac contractility was observed in TRIM32-KO mice compared with WT mice (Figure 5E). In parallel, compared with WT mice, the elevated mRNA expression levels of hypertrophic markers and fibrotic markers confirmed the further enhanced pathological remodelling in hearts of TRIM32-KO mice (Figures 5F and 3G). Collectively, TRIM32 deficiency did not lead to pathological alterations at baseline, but remarkably enhanced the susceptibility of the heart to cardiac hypertrophy and heart failure induced by pressure overload.

### TRIM32 inhibits the activation of Akt-dependent signalling pathways upon hypertrophic stresses

**Figure 6:**
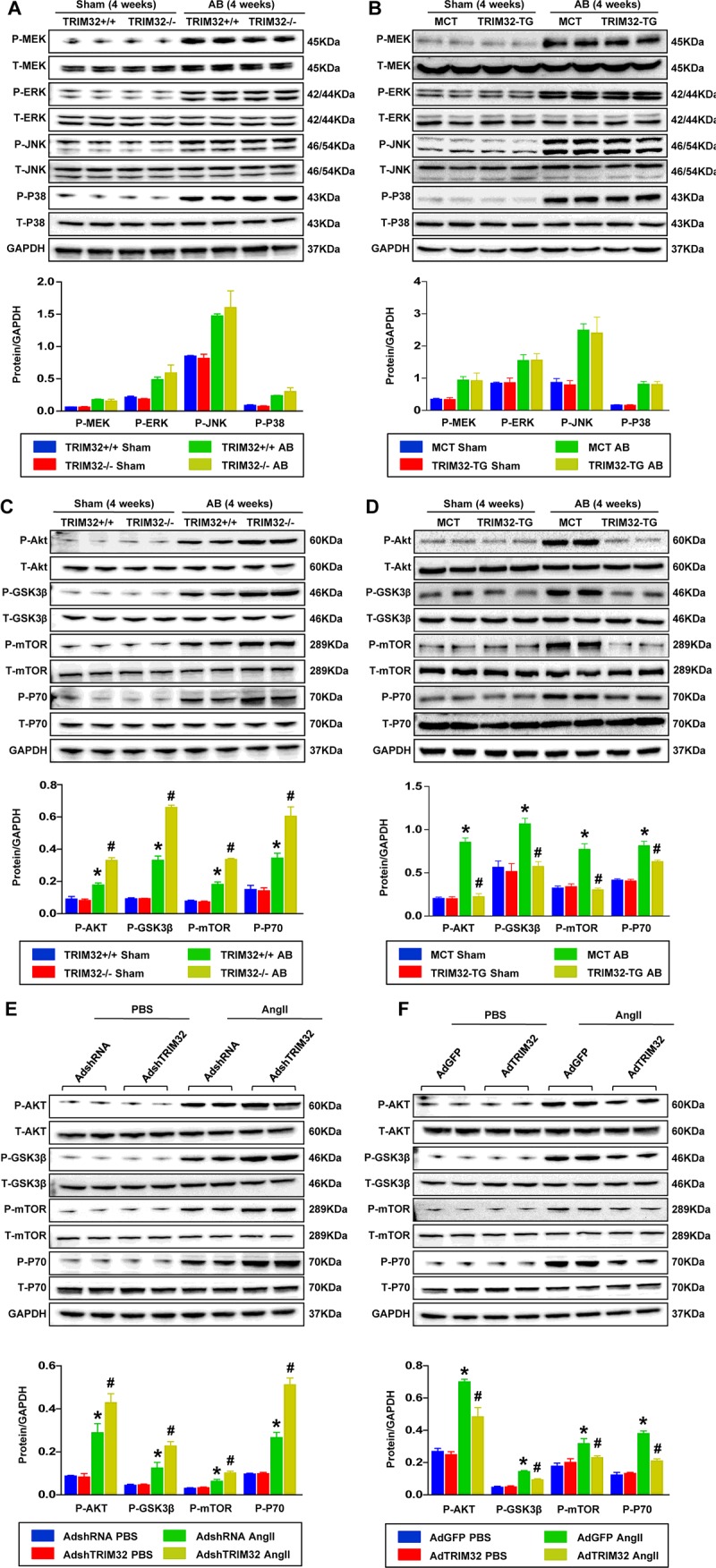
TRIM32 inhibits activation of Akt-dependent signalling pathways upon hypertrophic stresses (**A**) Representative Western blots (top) and quantitative results (bottom) of total (T-) and phospho- (P-) MAPK levels in the hearts of WT and TRIM32-KO mice 4 weeks after sham or AB surgery (*n*=4 mice per experiment group; *P<0.05 compared with WT/sham; #*P*<0.05 compared with WT/AB). (**B**) Representative Western blots (top) and quantitative results (bottom) of total (T-) and phospho- (P-) MAPK levels in the hearts of MCT and TRIM32-TG mice 4 weeks after sham or AB surgery (*n*=4 mice per experiment group; *P<0.05 compared with MCT/sham; #*P*<0.05 compared with MCT/AB). (**C**) Representative Western blots (top) and quantitative results (bottom) of total (T-) and phospho- (P-) Akt, GSK3β, mTOR and p70^S6K^ levels in the hearts of WT and TRIM32-KO mice 4 weeks after sham or AB surgery (*n*=4 mice per experiment group; *P<0.05 compared with WT/sham; #*P*<0.05 compared with WT/AB). (**D**) Representative Western blots (top) and quantitative results (bottom) of total (T-) and phospho- (P-) Akt, GSK3β, mTOR and p70^S6K^ levels in the hearts of MCT and TRIM32-TG mice 4 weeks after sham or AB surgery (*n*=4 mice per experiment group; *P<0.05 compared with MCT/sham; #*P*<0.05 compared with MCT/AB). (**E**) Representative Western blots (top) and quantitative results (bottom) of total (T-) and phospho- (P-) Akt, GSK3β, mTOR and p70^S6K^ levels in NRCMs infected with AdshRNA or AdshTRIM32 60 min after PBS or AngII administration (*n*=4 samples per experimental group; *P<0.05 compared with AdshRNA/PBS; #*P*<0.05 compared with AdshRNA/AngII). (**F**) Representative Western blots (top) and quantitative results (bottom) of total (T-) and phospho- (P-) Akt, GSK3β, mTOR and p70^S6K^ levels in NRCMs infected with AdGFP or AdTRIM32 60 min after PBS or AngII administration (*n*=4 samples per experimental group; *P<0.05 compared with AdGFP/PBS; #*P*<0.05 compared with AdGFP/AngII). GAPDH was used as a loading control. Results are means±S.D. from four independent experiments, and statistical analyses were performed using one-way ANOVA. Molecular masses are indicated in kDa in the Western blot panels.

To elucidate the mechanisms by which TRIM32 plays a cardioprotective role in cardiac hypertrophy and heart failure, we next examined the status of the MAPK and Akt signalling pathways, which are known to be involved in pathological cardiac hypertrophy [26–29]. Western blot assays revealed that there was no significant difference in the activation of MAPK signalling pathways, including MEK1/2, ERK1/2, JNK1/2 and p38, between WT and TRIM32-KO mice, or MCT and TG mice (Figures 6A and 6B). In contrast, TRIM32-deficient hearts showed dramatically elevated AB-mediated Akt activation (Figure 6C), whereas TRIM32-overexpressing hearts exhibited remarkably preserved levels of phosphorylated Akt 4 weeks after AB (Figure 6D). Similar alternations were observed in the phosphorylation of GSK3β, mTOR and p70^S6K^ (ribosomal p70 S6 kinase), which are downstream molecules of Akt (Figures 6C and 6D). However, the total expression of Akt and its downstream molecules were not altered among the groups tested. In accordance with the *in vivo* results, AngII-induced phosphorylation of Akt/GSK3β/mTOR/p70^S6K^ was aggravated in TRIM32-deficient cardiomyocytes, whereas the results were reversed in TRIM32-overexpressing cardiomyocytes (Figures 6E and 6F). According to these data, we speculate that TRIM32-mediated prevention of pathological cardiac hypertrophy is associated with blockade of Akt-dependent signalling.

### Blockage of Akt-dependent signalling pathways rescues pressure overload-induced cardiac abnormalities in TRIM32-KO mice

**Figure 7:**
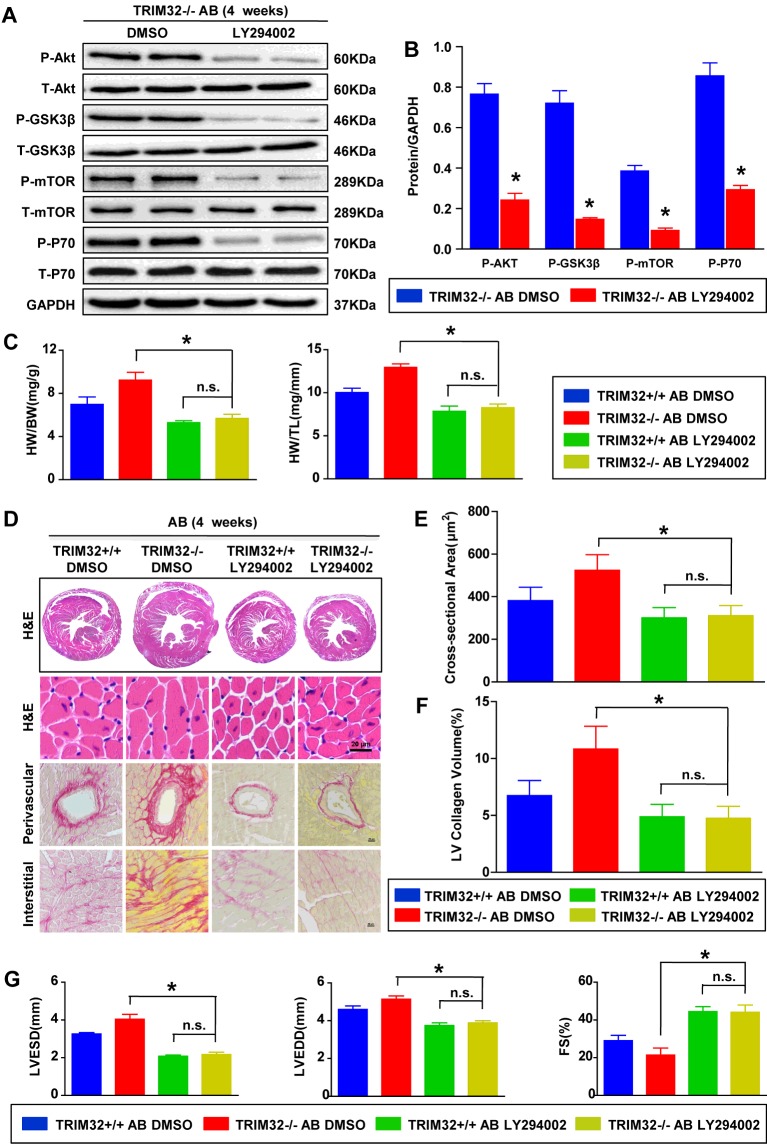
Blockage of Akt-dependent signalling pathways rescues pressure overload-induced cardiac abnormalities in TRIM32-KO mice (**A** and **B**) Representative Western blots (**A**) and quantitative results (**B**) of total (T-) and phospho- (P-) Akt-dependent signalling pathway protein levels in the hearts from LY294002- or DMSO-treated TRIM32-KO mice 4 weeks after AB surgery (*n*=4 mice per experiment group; GAPDH was used as a loading control). Molecular masses are indicated in kDa in (**A**). (**C**) Statistical results for HW/BW and HW/TL in the indicated groups (*n*=8–10 mice per experimental group). (**D**) Representative images of gross morphology, H&E staining and PSR staining from hearts in the indicated groups at 4 weeks after AB (*n*=5 mice per experimental group; scale bar, 20 μm for lower H&E staining and PSR staining). (**E**) Statistical results for the cardiomyocyte cross-sectional area in the indicated groups (*n*≥100 cells per experimental group). (**F**) Quantification of left ventricular collagen volume in the indicated groups (*n*≥25 fields per experimental group). (**G**) Measurements of LVESD, LVEDD and FS in the indicated groups (*n*=5 mice per experimental group). *P<0.05 compared with KO/AB/DMSO; n.s. indicates no significant difference. Results are means±S.D., and statistical analyses were performed using an unpaired Student's *t* test or one-way ANOVA.

To confirm the hypothesis that TRIM32 exerts a cardioprotective function by inhibiting Akt-dependent signalling pathways, TRIM32-KO mice were treated with the Akt-specific inhibitor LY294002 before being subjected to AB surgery. In contrast with DMSO-treated controls, Western blot analysis showed that LY294002 almost diminished the phosphorylation of Akt-dependent signalling pathways in hearts that underwent AB (Figures 7A and 7B). As predicted, LY294002 reversed hypertrophic deterioration in TRIM32-deficient hearts, as indicated by: (i) reduced HW/BW, HW/TL (Figure 7C), gross size of the heart (Figure 7D) and cardiomyocyte cross-sectional area (Figure 7E); (ii) decreased cardiac fibrosis in both the interstitial and perivascular area (Figures 7D and 7F); and (iii) improved cardiac function (Figure 7G) in TRIM32-KO mice treated with LY294002 compared with TRIM32-KO mice treated with PBS after AB. Collectively, these data indicate that the inhibitory effect of TRIM32 on pathological cardiac hypertrophy may be dependent, at least partially, on the regulation of Akt activity.

## DISCUSSION

In the present study, we aimed to delineate the potential function of TRIM32 in pressure overload-induced cardiac hypertrophy and heart failure using genetic manipulation of TRIM32 expression both *in vivo* and *in vitro*. TRIM32 expression significantly declined in failing human hearts, hypertrophic mouse hearts mediated by AB and cultured cardiomyocytes treated with AngII. Our data demonstrate that TRIM32 overexpression protected hearts from pathological hypertrophy, fibrosis, ventricular dilatation and dysfunction induced by chronic pressure overload. In contrast, reduced expression of TRIM32 was associated with an exaggerated hypertrophic response. Next, we validated that TRIM32 exerted its anti-hypertrophic effect through suppression of the Akt-dependent signalling pathways. To the best of our knowledge, we provide the first evidence that TRIM32 is a negative regulator of cardiac hypertrophy under pathological conditions.

TRIM32 is involved in a broad range of cellular processes including apoptosis [18,30], growth [17], proliferation [15], differentiation [31,32], protein degradation [20], the innate immune response [33] and mobility [17]. Consequently, its alterations are implicated in diverse pathological processes such as muscular dystrophy [9–13,19,20], oncogenesis [34,35], BBS11 [14], Alzheimer's disease [36] and psoriasis lesions [37]. However, the role of TRIM32 in cardiac hypertrophy and heart failure has not yet been established. In the present study, we observed that TRIM32 expression was progressively reduced from maladaptive hypertrophy to heart failure induced both by mechanical overload and by neurohumoral stimuli. These results strongly implied the involvement of TRIM32 in pathological cardiac hypertrophy. However, the limitation of the study was that it failed to elucidate the mechanisms underlying the down-regulation of TRIM32 expression in response to hypertrophic stresses. In contrast with the elevated TRIM32 expression observed in various pathological conditions, including independently derived tumours [35], Alzheimer's disease [36] and skeletal muscle subjected to hind-limb suspension [38], down-regulation of TRIM32 expression was seldom mentioned in the previous studies. Although the precise mechanisms are elusive, there is a potential explanation for the decreased TRIM32 content during the progression of cardiac hypertrophy. Interestingly, Ichimura et al. [39] indicated that 14-3-3 protein may promote TRIM32 stability by inhibiting autoubiquitylation and subsequent degradation via the proteasome. It is therefore tempting to speculate that hypertrophic stimuli may disrupt the positive regulation of 14-3-3 protein on TRIM32 stability. Thus TRIM32 would be reduced by self-association-based autoubiquitylation and subsequent proteasome degradation. However, additional studies are required to test our hypotheses.

On the basis of the decline in TRIM32 expression observed in hypertrophic hearts, we hypothesize that TRIM32 deficiency is associated with the progression of pathological cardiac hypertrophy, thus cardiac overexpression of TRIM32 may be protective. To test our hypotheses, gain-of-function and loss-of-function approaches were applied in the following experiments. In the present study, overexpression of TRIM32 in the heart blunted AB-induced hypertrophy and adverse remodelling, yet loss of TRIM32 had opposing effects. In parallel, *in vitro* experiments using NRCMs showed that TRIM32 knockdown significantly aggravated AngII-induced hypertrophy, whereas up-regulated TRIM32 expression repressed this hypertrophic response. Taken together, these data strongly support a cardioprotective role for TRIM32 in pathological hypertrophy.

Numerous intracellular signalling pathways participate in the regulation of cardiac hypertrophy [5]. First, we examined the effects of TRIM32 on the MAPK signalling pathways, which have been well documented to play an important role in cardiac hypertrophy [27]. However, our data indicate that the expression of TRIM32 has no influence on the expression or activity of MAPK signalling pathways in response to hypertrophic stimuli. These results exclude the possible involvement of MAPK signalling pathways in the cardioprotective role of TRIM32 upon chronic pressure overload. Recently, TRIM32 was reported to limit excessive growth in skeletal muscle by promoting plakoglobin–PI3K dissociation and subsequently inhibiting PI3K/Akt signalling [21]. As plakoglobin is highly expressed and functions as a component of the desmosome adhesion complex in cardiomyocytes, TRIM32 may exert an anti-hypertrophic effect in the heart in a similar manner. In response to hypertrophic stresses, Akt is activated by phosphorylated PI3K, and promotes hypertrophy largely via inhibitory effects on GSK3β and active effects on mTOR [40]. Inactive GSK3β relieves its inhibitory effects on a series of hypertrophic transcriptional effectors, including GATA4, β-catenin, c-Myc and NFAT (nuclear factor of activated T-cells), whereas active mTOR potentiates protein synthesis through phosphorylation of p70^S6K^. However, it remains controversial as to whether Akt is involved in physiological or pathological cardiac hypertrophy. The majority of the data affirmed that Akt1, a downstream mediator of IGF1R (insulin-like growth factor 1 receptor) and PI3Kα, plays a critical role in the induction of compensated heart growth and the preservation of systolic function upon hypertrophic stresses [41–45]. However, another study demonstrated that prolonged expression of activated Akt1 accounted for pathological hypertrophy with cardiac fibrosis, ventricular dilation and systolic dysfunction [46]. Likewise, Akt activated by PI3Kγ contributes to pathological hypertrophy during pressure overload or β-adrenergic receptor activation, but is dispensable for physiological growth [47–49]. Therefore the role of Akt in cardiac hypertrophy seems to be dependent on the type of stress and upstream mediator, as well as on the magnitude and duration of activation [50]. In the present study, activation of the Akt-dependent signalling pathways was dramatically blocked by TRIM32 overexpression, but exaggerated by TRIM32 deficiency in response to chronic hypertrophic stresses. These results suggest that the mechanisms underlying the cardioprotective effect of TRIM32 on the pathological cardiac hypertrophy could plausibly be dependent on inhibiting the prolonged activation of the Akt-dependent signalling pathways. Furthermore, we used the Akt-specific inhibitor LY294002 to reverse the activation of Akt induced by 4 weeks of AB in TRIM32-deficient mice. Consistent with the blunted Akt activation, LY294002 alleviated the hypertrophic growth and subsequent fibrosis of TRIM32-KO hearts subjected to chronic pressure overload. Altogether, these data support the concept that TRIM32 elicits its anti-hypertrophic actions largely by inhibition of Akt-dependent signalling pathways, and provides further evidence that Akt-dependent signalling pathways contribute to pathological hypertrophy in response to both biomechanical stress and neurohumoral mediators. However, the present study did not clearly elucidate the molecular mechanisms by which TRIM32 regulates the activation of Akt-dependent signalling pathways or the exact components of the Akt family which are responsible for the cardioprotective function of TRIM32. Thus additional studies are still required.

In conclusion, down-regulated TRIM32 expression accounts, at least in part, for hypertrophic remodelling of the heart upon chronic stresses. Furthermore, the results of the present study provide convincing evidence to support the concept that TRIM32 limits the development of cardiac hypertrophy, fibrosis and subsequent heart failure by interruption of the Akt-dependent signalling pathway. Thus these findings imply that TRIM32 represents a potential therapeutic target for the prevention of pathological cardiac hypertrophy.

## Abbreviations

AB: aortic banding
AdGFP: adenoviral vector encoding the *GFP* gene
AdshRNA: adenoviral vector encoding scrambled shRNA sequence
AdshTRIM32: adenoviral vector encoding the TRIM32 shRNA sequence
AdTRIM32: adenoviral vector encoding *TRIM32* cDNA
AngII: angiotensin II
ANP: atrial natriuretic peptide
BBS11: Bardet–Biedl syndrome type 11
BNP: brain natriuretic peptide
Cas9: CRISPR-associated 9
CRISPR: clustered regularly interspaced short palindromic repeats
CTGF: connective tissue growth factor
DCM: dilated cardiomyopathy
DMEM: Dulbecco's modified Eagle's medium
ERK: extracellular-signal-regulated kinase
FS: fractional shortening
GAPDH: glyceraldehyde-3-phosphate dehydrogenase
GSK3β: glycogen synthase kinase 3β
H&E: haematoxylin and eosin
HW/BW: heart weight/body weight ratio
HW/TL: heart weight/tibia length ratio
JNK: c-Jun N-terminal kinase
KO: knockout
LV: left ventricle
LVEDD: LV end-diastolic diameter
LVESD: LV end-systolic diameter
MAPK: mitogen-activated protein kinase
MCT: α-MHC-MerCreMer mice with tamoxifen administration
MEK: MAPK/ERK kinase
β-MHC: β-myosin heavy chain
mTOR: mammalian target of rapamycin
NRCM: neonatal rat cardiomyocyte
p70^S6K^: ribosomal p70 S6 kinase
PI3K: phosphoinositide 3-kinase
PSR: Picrosirius Red
sgRNA: single guide RNA
TG: transgenic
TRIM32: tripartite motif 32
WGA: wheatgerm agglutinin
WT: wild-type
